# Transformers meet CNNs for insights into breast mass classification from histopathological images

**DOI:** 10.3389/frai.2026.1770667

**Published:** 2026-03-19

**Authors:** Vatsala Anand, Ajay Khajuria

**Affiliations:** 1 Department of Computer Science and Engineering, Akal University, Bathinda, Punjab, India; 2 Department of Chemistry, Akal University, Bathinda, Punjab, India

**Keywords:** breast tumors, ConvNeXt V2, histopathology, mammography, medical image analysis, Swin Transformer V2, vision transformers, classification

## Abstract

**Introduction:**

Breast cancer remains one of the leading causes of cancer-related deaths among women worldwide, highlighting the critical need for accurate histopathological diagnosis and reliable decision-support systems to improve diagnostic sensitivity and reduce false-negative outcomes.

**Methods:**

In this research, a deep learning-based approach for binary classification of breast cancer into benign and malignant categories utilizing histopathological images is presented. A dataset comprising 10,000 high-resolution histopathology images was used to evaluate the execution of two vision models: Swin Transformer V2 and ConvNeXt V2. Swin Transformer V2, a progressive vision transformer with shifted window self-attention, and ConvNeXt V2, a modern convolutional neural network motivated by transformer plans, were fine-tuned and tested for their adequacy in feature representation and classification accuracy.

**Results:**

The experimental results demonstrate that Swin Transformer V2 consistently outperforms ConvNeXt V2 across all evaluation metrics, achieving a peak classification accuracy of 0.985, which reflects its superior capability in capturing subtle morphological and contextual variations in histopathological tissues.

**Discussion:**

The attention-driven global feature modeling in Swin Transformer V2 enables more discriminative representations compared to convolutional inductive biases, particularly for complex cellular patterns. These findings suggest that transformer-based architectures offer significant advantages over modern CNNs for histopathological breast cancer classification, and they hold substantial potential for advancing computer-aided diagnosis systems in digital pathology. The comparative insights provided in this study can guide the selection of robust deep learning models for scalable and reliable clinical decision-support systems.

## 1 Introduction

Breast cancer is one of the most common types of cancer affecting women around the world and continues to be a major cause of death from cancer, even with ongoing improvements in screening and treatment methods. The World Health Organization emphasizes that early and precise diagnosis is essential for increasing the chances of survival for patients and lowering the overall impact of the disease. While there are several ways to diagnose breast cancer, examining breast tissue through histopathological analysis is still considered the best standard in clinical practice. This method gives detailed information about the structure of the tissue, which is important for confirming whether cancer is present and for deciding the best course of treatment. However, looking at histopathology slides by hand takes a lot of time and effort, and it relies heavily on the skill and experience of the pathologist involved. This can lead to differences in how different doctors interpret the same results, known as inter- and intra-observer variability, which may result in inconsistent diagnoses, particularly in cases that are unclear or complex (Springenberg et al., 2023).

To tackle these problems, computer-aided diagnosis (CAD) systems have become a helpful tool for pathologists. These systems offer objective, reliable, and repeatable evaluations. In recent years, deep learning methods have become the main part of modern CAD systems, especially for analyzing histopathological images. This is because deep learning can automatically learn important features from raw image data, without needing manually designed features (Shiri et al., 2024). Convolutional neural networks (CNNs), like VGG, ResNet, DenseNet, and EfficientNet, have shown great results in detecting and classifying breast cancer by learning spatial patterns at different levels (Fiaz et al., 2024; Kaczmarek et al., 2025; Hossain et al., 2025). However, traditional CNNs depend a lot on local areas and specific assumptions about how data works, which might stop them from understanding long-distance relationships and the overall structure of tissues, both of which are very important in histopathological analysis.

Recently, Vision Transformers (ViTs) have become very popular as a strong option compared to CNNs in computer vision tasks. They use self-attention mechanisms to understand relationships across different parts of an image, which helps them see both small details and bigger structures (Jiang et al., 2024). Among different transformer models, the Swin Transformer uses a special design called shifted window self-attention to create a more efficient and organized structure, which works well with high-quality medical images (Javed et al., 2025). The newer version, Swin Transformer V2, improves training stability and makes it easier to handle large datasets, solving problems like sensitivity to image size and attention saturation that often happen in big histopathological studies.

At the same time, modern CNN structures have been greatly redesigned with ideas from transformers. ConvNeXt V2, developed by Meta AI, is a new type of convolutional network that keeps the efficient parts of CNNs while using some strategies from transformers, like bigger kernels, layer normalization, and better training goals. A big set of 10,000 high-quality histopathology images is used to make sure the testing is strong and fair. Both models are trained in the same way, and their results are checked using several important medical measurements, such as accuracy, precision, recall, F1-score, and ROC analysis. The primary objective of this research is to evaluate and compare the classification performance of these two state-of-the-art models in the context of breast cancer diagnosis. Our results demonstrate that the Swin Transformer V2 outperforms ConvNeXt V2 across several evaluation metrics, highlighting the potential of vision transformers for advancing diagnostic accuracy in digital pathology.

**Figure 1:**
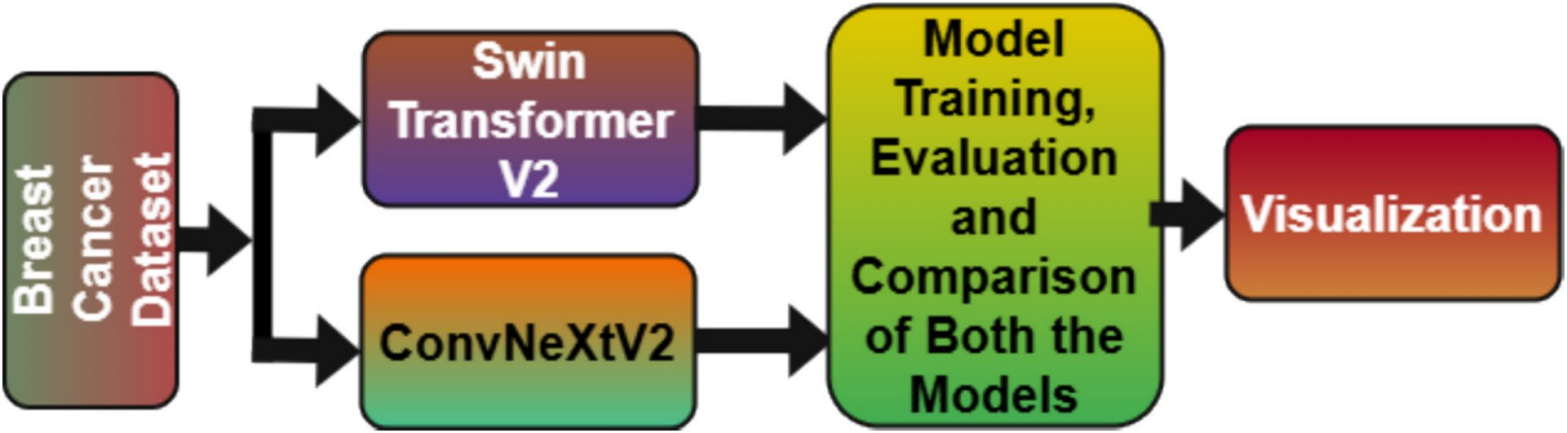
Theme diagram of proposed study.

Figure 1 shows the theme depiction of the proposed study that includes dataset description, two models’ description and after that, training, evaluation, comparison, and visualization of the models used in the study.

## 2 Literature review

Breast cancer classification has been a critical area of research, with numerous studies exploring advanced techniques to improve diagnostic accuracy and early detection. Shan et al. (2012) had proposed a breast cancer segmentation method using a database of 120 images. The proposed method improves the true positive rate from 84.9–92.8%. Torbati et al. (2014) had proposed a method for the segmentation of breast cancer. They had worked on X-Ray, CT and MR images. Cheng et al. (2016) had performed a study for the segmentation of breast cancer into benign and malignant tumors. (Huynh et al., 2016) had performed an image fusion method with the combination of different architectures. They had worked using 1,687 tumor images and had obtained the value of accuracy as 91.10%. Fujioka et al. (2019) have presented a distinction of benign and malignant breast tumors using 480 images of breast masses. They had obtained the value of sensitivity as 0.958. Yap et al. (2017) had presented breast cancer segmentation using ultrasound images. They had used two datasets, having 306 and 163 images. Huang et al. (2020) had presented breast cancer segmentation using BUS images containing 320 cases. They had obtained the F1-score of 89.87%. Lee et al. (2020) had presented an attention channel module for the segmentation of breast cancer. Building upon these earlier works, this study investigates the viability of progressed deep learning architectures—specifically Swin Transformer V2 and ConvNeXt V2 pointing to improve diagnostic accuracy through predominant feature representation and classification performance. Wakili et al. (2025) presented a hybrid breast cancer histopathology classification framework that integrates DenseNet for deep feature extraction and the Flower Pollination Algorithm for optimized feature selection. High classification accuracies are achieved on BreakHis and BACH datasets. However, the model lacks interpretability and is validated only on benchmark datasets, limiting clinical applicability.

Pradeepa et al. (2025) proposed a hybrid deep learning architecture combining EfficientNetV2 and an attention-enhanced GRU to capture spatial and sequential dependencies in histopathological images. Evaluated on BreakHis and Camelyon17 datasets at fixed magnification, the model achieves strong performance, though its scope is limited to patch-level analysis and lacks multi-resolution whole-slide integration. A hybrid transfer learning framework using fine-tuned Xception features with traditional classifiers (SVC and Random Forest) is introduced for breast cancer histopathology classification. The method demonstrates robustness across magnification levels on BreakHis and BHID datasets. However, it remains patch-based, relies on transfer learning without contextual modeling, and lacks large-scale clinical validation (Gupta et al., 2025). Yusuf et al. (2025) proposed an Enhanced Shallow Convolutional Neural Network for multi-class breast cancer histopathology classification, emphasizing low computational cost and magnification-aware learning. While achieving competitive accuracy across multiple magnifications on the BreakHis dataset, the shallow architecture may miss complex pathological patterns and has limited generalizability beyond benchmark datasets. Pandey et al. (2025) employed a transfer learning–based VGG-16 feature extractor combined with classical machine learning classifiers, with and without PCA, for breast cancer image classification. Excellent binary classification accuracy is reported on the BreakHis dataset. However, reliance on PCA, lack of end-to-end learning, and single-dataset evaluation constrain scalability and real-world deployment. Table 1 shows the related work findings.

**Table 1:** Related work findings.

References	Methods used	Outcome	Limitations
Shan et al. (2012)	Fully automated breast ultrasound (BUS) lesion segmentation framework using automatic ROI generation. Artificial Neural Network (ANN).	TP rate increased to 92.8%, Similarity rate to 83.1%, FP rate reduced to 12.0%	Evaluation is performed on a small dataset, limiting statistical generalization. The approach relies on handcrafted features, which may restrict adaptability compared to deep end-to-end learning methods. Applicability to other imaging modalities or large-scale clinical settings was not investigated.
Torbati et al. (2014)	Proposed a modified Self-Organizing Map (MA-SOM)–based medical image segmentation method. A 2D Discrete Wavelet Transform (DWT) is used to construct the feature space. Evaluated on breast ultrasound (BUS) images as well as CT and MRI head images, and compared with SOM and ISNN-based methods.	Reported TP rate >93% for white and gray matter, FP ratio <0.8%, and superior Jaccard, Rogers–Tanimoto indices compared to SOM.	Evaluation was conducted on small datasets (30 BUS images and limited MRI samples), which may restrict generalizability. Performance degradation was observed for CSF regions under high noise conditions. The method is not fully automated and relies on handcrafted features, limiting scalability and integration with modern end-to-end deep learning CAD systems.
Cheng et al. (2016)	Deep learning–based computer-aided diagnosis (CADx) framework using a Stacked Denoising Auto-Encoder (SDAE) for automatic and robust feature learning. Performance evaluated using 10 × 10-fold cross-validation and compared with two conventional CADx algorithms.	Demonstrated a significant performance improvement over conventional CADx methods in differentiating benign and malignant lesions. Showed strong noise tolerance, robust feature representation, and Improved diagnostic reliability across multiple imaging modalities	Performance analysis is limited to comparison with only two conventional CADx algorithms. Dataset size and diversity are not explicitly discussed, which may affect generalizability. The approach focuses on binary classification and does not address multi-class diagnosis or real-time clinical deployment. Interpretability of learned features is not explored.
Huynh et al. (2016)	Utilized transfer learning by extracting features from pre-trained deep CNNs and training Support Vector Machine (SVM) classifiers. Compared CNN-extracted features with human-designed (handcrafted) tumor features. Evaluated on a breast ultrasound dataset of 1,125 cases and 2,393 ROIs using five-fold case-based cross-validation.	Achieved competitive diagnostic performance with reduced computational cost. For non-malignant vs. malignant classification, both CNN-based and handcrafted-feature-based SVMs achieved an AUC of 0.90. For benign vs. malignant classification, CNN-feature-based SVM achieved a higher AUC of 0.88 compared to 0.85 using human-designed features.	Performance is limited to binary classification tasks and relies on pre-trained CNN representations without end-to-end fine-tuning. Improvements over handcrafted features are modest in some tasks. The study focuses on ROI-level analysis and does not address full-image or real-time clinical deployment. Interpretability of CNN-derived features is not explicitly discussed.
Fujioka et al. (2019)	Applied a deep convolutional neural network (GoogLeNet architecture) for binary classification of benign vs. malignant breast masses from ultrasound images. Model trained on retrospectively collected ultrasound data and evaluated against interpretations from three radiologists using sensitivity, specificity, accuracy, and AUC metrics.	Demonstrated high diagnostic performance, achieving sensitivity of 0.958, specificity of 0.925, accuracy of 0.925, and AUC of 0.913. The CNN showed equal or superior performance compared to radiologists, whose AUC ranged from 0.728 to 0.845, highlighting the potential of deep learning as a reliable decision-support tool.	Study is based on a relatively small, single-center retrospective dataset, which may limit generalizability. The analysis is restricted to binary classification, without lesion segmentation or multi-class diagnosis. Model interpretability and real-time clinical integration were not addressed.
Yap et al. (2017)	Investigated three deep learning approaches for breast ultrasound (BUS) lesion detection: patch-based LeNet, U-Net, and transfer learning with pretrained FCN-AlexNet. Performance compared with four state-of-the-art conventional methods: Radial Gradient Index, Multifractal Filtering, Rule-based Region Ranking, and Deformable Part Models (DPM). Evaluated on two ultrasound datasets acquired using different imaging systems.	Deep learning methods demonstrated overall superior detection performance across both datasets in terms of True Positive Fraction (TPF), false positives per image, and F-measure. FCN-AlexNet achieved the best results on Dataset A, while patch-based LeNet performed best on Dataset B. The study highlighted the adaptability and robustness of learning-based lesion detection methods across heterogeneous datasets.	Performance depends on the availability of labeled training data, including negative samples. Evaluation is limited to 2D ultrasound lesion detection only, without downstream segmentation or classification. Dataset sizes are relatively small, and multi-center validation is not explored. The lack of a unified public benchmark remains a challenge.
Huang et al. (2020)	Proposed a supervised breast ultrasound (BUS) tumor segmentation method based on semantic classification and superpixel merging. ROI is manually selected, followed by image enhancement (histogram equalization, bilateral filtering, pyramid mean shift). SLIC superpixels are generated, semantic features extracted, and a Bag-of-Words (BoW) model constructed. Initial classification is performed using a Backpropagation Neural Network (BPNN) and refined using K-Nearest Neighbors (KNN) reclassification.	Achieved competitive segmentation performance on a BUS dataset of 320 cases, producing tumor contours close to hand-labeled annotations. Reported an average F1-score of 89.87% ± 4.05% and favorable TP/FP rates compared with five existing approaches. Demonstrated that semantic superpixel classification can effectively guide tumor boundary delineation.	The method is not fully automated, requiring manual ROI selection by the operator. Performance strongly depends on classifier accuracy and dataset-specific training, limiting generalization across imaging devices. While effective, results are inferior to fully supervised deep learning models (e.g., FCN) trained with pixel-level annotations. Scalability to large datasets and real-time clinical deployment was not evaluated.
Lee et al. (2020)	Proposed a novel semantic segmentation network incorporating a channel attention module with Multiscale Grid Average Pooling (MSGRAP) to improve breast cancer segmentation in ultrasound images. The attention module captures both global and local spatial information, overcoming limitations of standard convolution. Network architecture was optimized through ablation studies and evaluated against FCN, SegNet, U-Net, and other state-of-the-art deep learning models.	Demonstrated superior segmentation performance compared to existing deep learning approaches on a public BUS dataset. The proposed channel attention module significantly enhanced segmentation accuracy, showing better semantic consistency and boundary delineation of breast cancer regions.	Performance remains dependent on the quality and diversity of annotated training data. Like other deep learning approaches, the model requires pixel-level ground truth, increasing annotation cost. Generalization to ultrasound images from different acquisition devices or clinical settings was not extensively analyzed.
Wakili et al. (2025)	Hybrid framework integrating Dense Convolutional Network (DenseNet) for hierarchical feature extraction with Flower Pollination Algorithm (FPA) for optimized feature selection on histopathological images (BreakHis and BACH datasets).	Achieved high classification accuracy of 99.32% on BreakHis and 96% on BACH datasets, outperforming existing state-of-the-art methods. Demonstrated improved generalization, reduced computational complexity, and enhanced diagnostic performance	Model interpretability remains limited due to the black-box nature of deep learning. Validation is restricted to benchmark datasets; real-world clinical deployment and large-scale multi-center validation are not explored. Computational cost of hybrid optimization may increase training time.
Pradeepa et al. (2025)	Hybrid deep learning framework combining EfficientNetV2 for multi-scale spatial feature extraction with a Gated Recurrent Unit (GRU) enhanced by an attention mechanism to capture spatial and contextual dependencies in breast cancer histopathology images. Includes preprocessing with normalization, resizing, ImageNet-based standardization, and Otsu’s thresholding. Evaluated on BreakHis and Camelyon17 datasets at 200 × magnification.	Achieved superior performance over AlexNet, DenseNet, MobileNetV3, and EfficientNet. Reported 98.15% precision, 95.68% recall, 96.82% F1-score, 96% specificity, 93.99% IoU, and 95.72% accuracy. Demonstrated effective spatial–sequential feature learning, good generalization across datasets, fast inference, and improved interpretability via attention mechanisms.	Model evaluation is primarily limited to patch-level classification and a fixed 200 × magnification, restricting applicability to whole-slide image (WSI) analysis. Absence of data augmentation may limit robustness to morphological variability. Multi-resolution and real-time WSI integration are not explored.
Gupta et al. (2025)	Hybrid deep transfer learning framework using fine-tuned Xception for feature extraction combined with traditional classifiers: Support Vector Classifier (SVC) and Random Forest Classifier (RFC). Includes pre-processing, class balancing, and hyper-parameter optimization. Evaluated and compared with LR, K-NN, AdaBoost, and state-of-the-art methods on BreakHis and BHID datasets across multiple image magnification levels (40 × −400×).	XSV and XRF models outperformed conventional ML classifiers. XSV achieved accuracies of 89.26, 85.87, 90.17, and 88.98% on BreakHis at 40×, 100×, 200×, and 400 × magnifications, respectively. XRF achieved accuracies up to 88.98%. On BHID (40×), XSV and XRF reached 87.35 and 87.29%, respectively. Demonstrated robustness across magnification levels and effectiveness as a computer-aided diagnostic system.	Performance remains dependent on dataset size and class imbalance, particularly limited malignant samples. Feature learning relies on transfer learning without explicit spatial or contextual modeling. The approach is restricted to patch-level classification and does not address whole-slide image (WSI) analysis or real-time clinical deployment. Large-scale multi-center validation is not explored.
Yusuf et al. (2025)	Proposed an Enhanced Shallow Convolutional Neural Network (ES-CNN) designed for multi-class breast cancer histopathology image classification. The architecture is tailored to account for magnification and patient dependence, using fewer convolutional layers and weights to reduce computational complexity. Evaluated on the BreakHis dataset across four magnification levels (40×, 100×, 200×, 400×).	Achieved multi-class classification accuracies of 96% (400×), 95% (200×), 98% (100×), and 96% (40×). Demonstrated significantly reduced training time and computational utilization compared to deep CNNs, while maintaining competitive or superior performance relative to state-of-the-art deep architectures.	Performance evaluation is limited to the BreakHis dataset, which may restrict generalizability. Shallow architecture, while computationally efficient, may limit the model’s ability to capture highly complex or subtle pathological patterns. The approach does not leverage transfer learning or advanced interpretability mechanisms, and real-world clinical deployment was not investigated.
Pandey et al. (2025)	Transfer learning–based CNN feature extractor (VGG-16) combined with traditional machine learning classifiers (SVM, Decision Tree, KNN, LDA, Boosted Tree). Two strategies evaluated: with and without PCA for feature reduction. Experiments conducted on the BreaKHis dataset (7,909 H&E-stained images from 82 patients).	Achieved very high classification performance. SVM with PCA reached 99.5% accuracy with significantly reduced execution time, while Decision Tree without PCA achieved 99.4% accuracy. Demonstrated effective trade-off between accuracy and computational efficiency and outperformed several deep learning baselines across metrics such as accuracy, precision, recall, and ROC.	Reliance on PCA-based feature selection may limit exploration of more advanced wrapper or embedded feature selection methods. Evaluation is restricted to a single dataset and binary classification, limiting generalizability. Spatial context learning and end-to-end deep optimization are not fully exploited. Clinical deployment and multi-center validation are not addressed.

The proposed model’s performance on two mammography datasets: Mammography Breast Cancer Detection (Authors, n.d.) and DDSM (CBIS-DDSM, 2017) has been observed. Each dataset has a different number of classes and images, which affects the results. The Mammography Breast Cancer Detection dataset has two types of images — benign and malignant. There are 53.5 thousand benign images and only 1,158 malignant ones. That means there are way more benign images than malignant ones. Even with this big difference, the models did well. They had a precision of 85.43%, sensitivity of 89.34%, F1-score of 91.45%, and an overall accuracy of 95.23%. These high numbers show the models can handle big datasets and tell the difference between benign and malignant images, which is very important for doctors. The DDSM dataset has three types of images: normal, benign, and malignant, making a total of 2,620 images.

The models performed with a precision of 84.45%, sensitivity of 86.36%, F1-score of 89.10%, and accuracy of 93.02%. These numbers are a bit lower than the ones from the other dataset, but they are still strong, especially since there are more classes and fewer images. Looking at both datasets, the models performed well in different situations. They did better on the bigger, simpler dataset. These results show that the models are reliable and can be used in real-world situations for detecting breast cancer.

The mammography datasets discussed in this section are presented for contextual and comparative purposes only. These datasets were not used for independent training or testing within the scope of the present study. No additional preprocessing, splitting, or experimental evaluation was performed on these datasets. The discussion is intended to situate our findings within the broader literature rather than to claim external validation.

### 2.1 Research gap

Table 2 presents the research gap obtained in the related work performed by different researchers in the same field.

**Table 2:** Research gap in related work.

References	Technique	Merits	Demerits
Shan et al. (2012)	Fully automated BUS lesion segmentation using ANN with automatic ROI generation	Improved TP rate and similarity while reducing FP rate; early attempt toward automation	Small dataset limits generalization; dependence on handcrafted features restricts adaptability; lacks robustness across devices and imaging modalities; no end-to-end deep feature learning
Torbati et al. (2014)	Modified SOM-based segmentation using DWT features	Good TP and FP performance; evaluated across BUS, CT, and MRI modalities	Very limited dataset size; manual intervention required; performance degrades under noise; relies on handcrafted features; not scalable to modern deep learning CAD systems
Cheng et al. (2016)	Stacked Denoising Auto-Encoder (SDAE) for CADx	Robust feature learning and noise tolerance; outperforming conventional CADx methods	Limited comparison baseline; dataset diversity not reported; binary diagnosis only; interpretability and real-time deployment not addressed
Huynh et al. (2016)	Transfer learning from pretrained CNNs with SVM classifier	Reduced computational cost; improved AUC over handcrafted features	No end-to-end learning; gains over handcrafted features are modest; ROI-based analysis only; lacks segmentation and clinical workflow integration
Fujioka et al. (2019)	GoogLeNet CNN for BUS lesion classification	Performance comparable or superior to radiologists; high diagnostic accuracy	Single-center and retrospective dataset; binary classification only; no lesion segmentation or explainability; limited evaluation of clinical usability
Yap et al. (2017)	Patch-based LeNet, U-Net, and FCN-AlexNet for BUS lesion detection	Demonstrated superiority of DL methods over conventional detectors; adaptable across datasets	Focused only on lesion detection (not segmentation or diagnosis); small datasets; lack of unified benchmark; no end-to-end CAD framework
Huang et al. (2020)	Superpixel-based semantic classification with BPNN and KNN	Competitive segmentation accuracy; weakly supervised alternative to FCN	Manual ROI selection required; performance highly dataset-dependent; inferior to fully supervised DL; limited scalability and clinical automation
Lee et al. (2020)	Channel attention with MSGRAP for BUS semantic segmentation	Improved boundary delineation; better global–local feature integration	Requires pixel-level annotations; annotation cost is high; generalization across scanners not validated; lacks lightweight or real-time design
Wakili et al. (2025)	DenseNet with Flower Pollination Algorithm for histopathology classification	Very high accuracy on BreakHis and BACH; optimized feature selection	Black-box nature limits interpretability; validated only on benchmark datasets; computationally intensive optimization; no clinical deployment analysis
Pradeepa et al. (2025)	EfficientNetV2 with GRU and attention for histopathology images	Strong spatial–sequential feature modeling; good cross-dataset performance	Patch-level analysis only; fixed magnification; no WSI-level modeling; limited robustness analysis
Gupta et al. (2025)	Deep transfer learning with classical ML classifiers	Robust across magnifications; effective CAD assistance	No contextual or spatial dependency modeling; patch-level only; class imbalance issues; no real-time or WSI-level integration
Yusuf et al. (2025)	Shallow CNN for multi-class histopathology classification	Low computation cost; high accuracy; fast training	Limited feature depth; evaluated on one dataset; lacks transfer learning and interpretability; clinical translation unaddressed
Pandey et al. (2025)	VGG-16 features with ML classifiers and PCA	Extremely high accuracy with reduced execution time	Binary classification only; reliance on PCA; no end-to-end learning; spatial feature learning and multi-center validation missing

### 2.2 Novelty of work

- *Comparative assessment of advanced architectures*: This study presents a comprehensive comparison between two models, namely Swin Transformer V2 and ConvNeXt V2 for the binary classification of breast cancer using histopathological images, offering new insights into the applicability of transformer-based models versus modernized CNNs in medical imaging.
- *Application to a large-scale histopathology dataset*: A curated dataset comprising 10,000 high-resolution histopathological images was utilized, contributing to the growing body of work that leverages large datasets for robust and generalizable model performance in breast cancer diagnosis.
- *Demonstration of Swin Transformer V2’s superiority in medical context*: The experimental results show that Swin Transformer V2 significantly outperforms ConvNeXt V2 in key classification metrics, establishing its efficacy for learning both local and global tissue structures in complex medical imagery.
- *Insight into the role of architectural inductive bias*: The study highlights how the architectural differences—namely, the hierarchical attention mechanism in Swin Transformer V2 versus the convolutional locality of ConvNeXt V2—impact performance on histopathological data, contributing to the understanding of design choices in deep learning for digital pathology.

By achieving high classification performance, especially with Swin Transformer V2, this work demonstrates the potential for vision transformer-based CAD systems to support pathologists in early detection and diagnosis of breast cancer, paving the way for integration into real-world diagnostic workflows.

## 3 Proposed methodology

**Figure 2:**
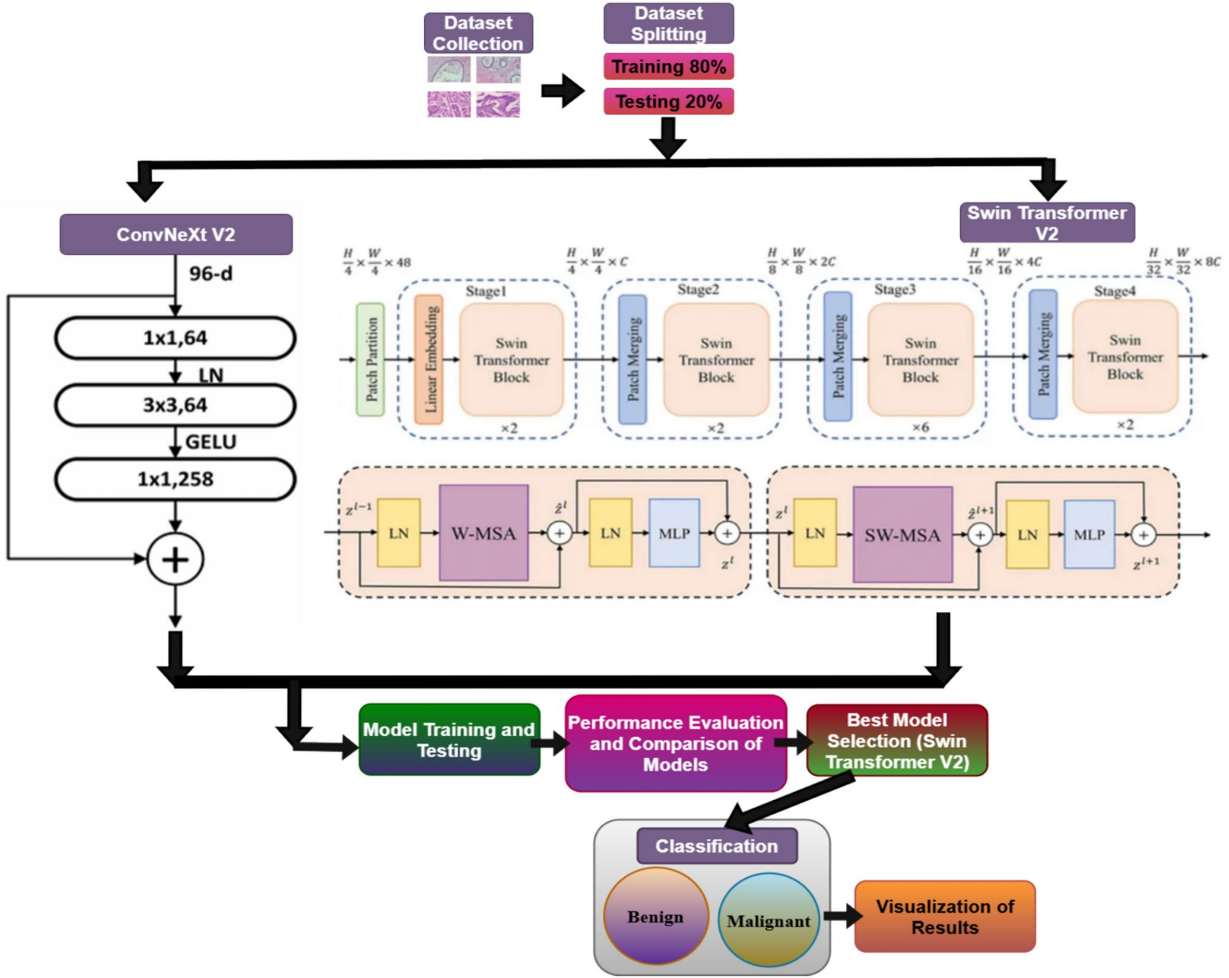
Proposed model overview.

This section outlines the technique adopted for breast cancer classification as shown in Figure 2. The study begins with a detailed description of the dataset used, followed by the implementation of two advanced deep learning models: Swin Transformer V2 and ConvNeXt V2. These models are prepared and evaluated utilizing a strong training system to ensure a suitable comparison of their execution. The final stage incorporates a comprehensive assessment of their classification capabilities, highlighting the qualities and limitations of each approach in accurately diagnosing breast cancer.

Both Swin Transformer V2 and ConvNeXt V2 were initialized using publicly available pre-trained weights trained on the ImageNet-1 K dataset. Transfer learning was employed to leverage general-purpose visual feature representations learned from large-scale natural image data before adaptation to the histopathological domain. For the breast cancer classification task, the original ImageNet classification heads were removed from both architectures. A new task-specific fully connected (linear) layer was appended to each backbone to accommodate binary classification (benign vs. malignant).

During training, the entire network, including both the pretrained backbone and the newly added classifier head, was fine-tuned on the histopathological dataset. This end-to-end optimization enabled the models to adapt high-level semantic representations to domain-specific morphological and cellular features relevant to breast cancer diagnosis.

### 3.1 Dataset description

A curated collection of medical images is included in the dataset for breast cancer classification (Authors, n.d.). This study utilizes the publicly available BreaKHis (Breast Cancer Histopathological Image Classification) dataset. The dataset comprises a total of 10,000 images collected from 82 patients using Hematoxylin and Eosin (H&E) staining. Images were acquired under four different magnification factors: 40×, 100×, 200×, and 400 ×.

**Figure 3:**
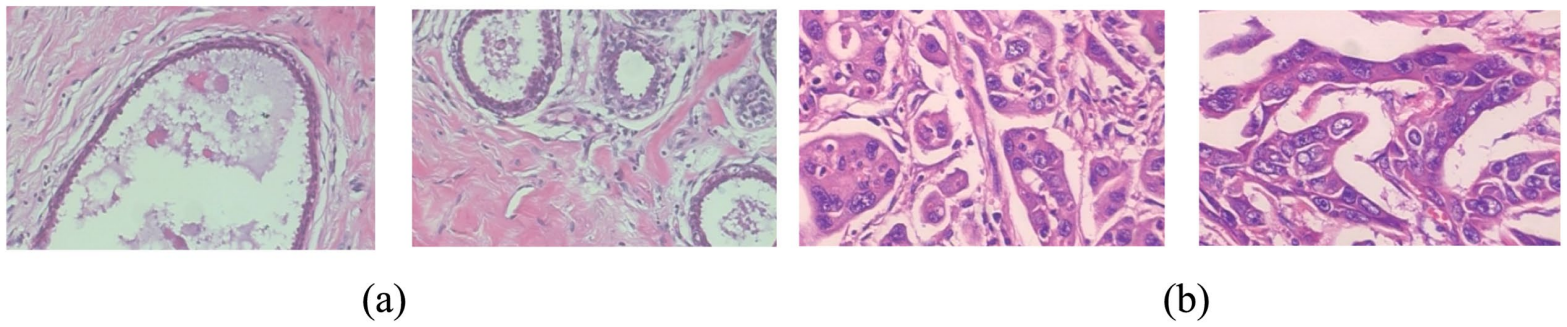
Dataset samples **(a)** benign and **(b)** malignant.

The dataset contains two primary diagnostic categories: benign and malignant tumors. These labels were assigned based on histopathological examination by expert pathologists at the time of data collection. As BreaKHis is a curated and publicly validated dataset widely used in medical image analysis research, label reliability is ensured through expert annotation at the source institution. No relabeling or manual modification of labels was performed in this study. The dataset was created to partition breast cancers into two distinct categories, as shown in Figure 3. The advancement and assessment of calculations that utilize deep learning for breast cancer identification and categorization can be helped by this dataset, which bears significance for the progression of the area of analysis of medical images. The dataset is made up of 10,000 medical images that have been systematically organized into two categories: benign tumors and malignant tumors. A balanced distribution that ensures robust model training and evaluation is ensured by the fact that each group is accompanied by 4,696 benign images and 5,304 malignant images.

### 3.2 Different classification models

In this study, training and evaluation is performed using Swin Transformer V2 and ConvNeXt V2 architectures.

#### 3.2.1 Swin Transformer V2

**Figure 4:**
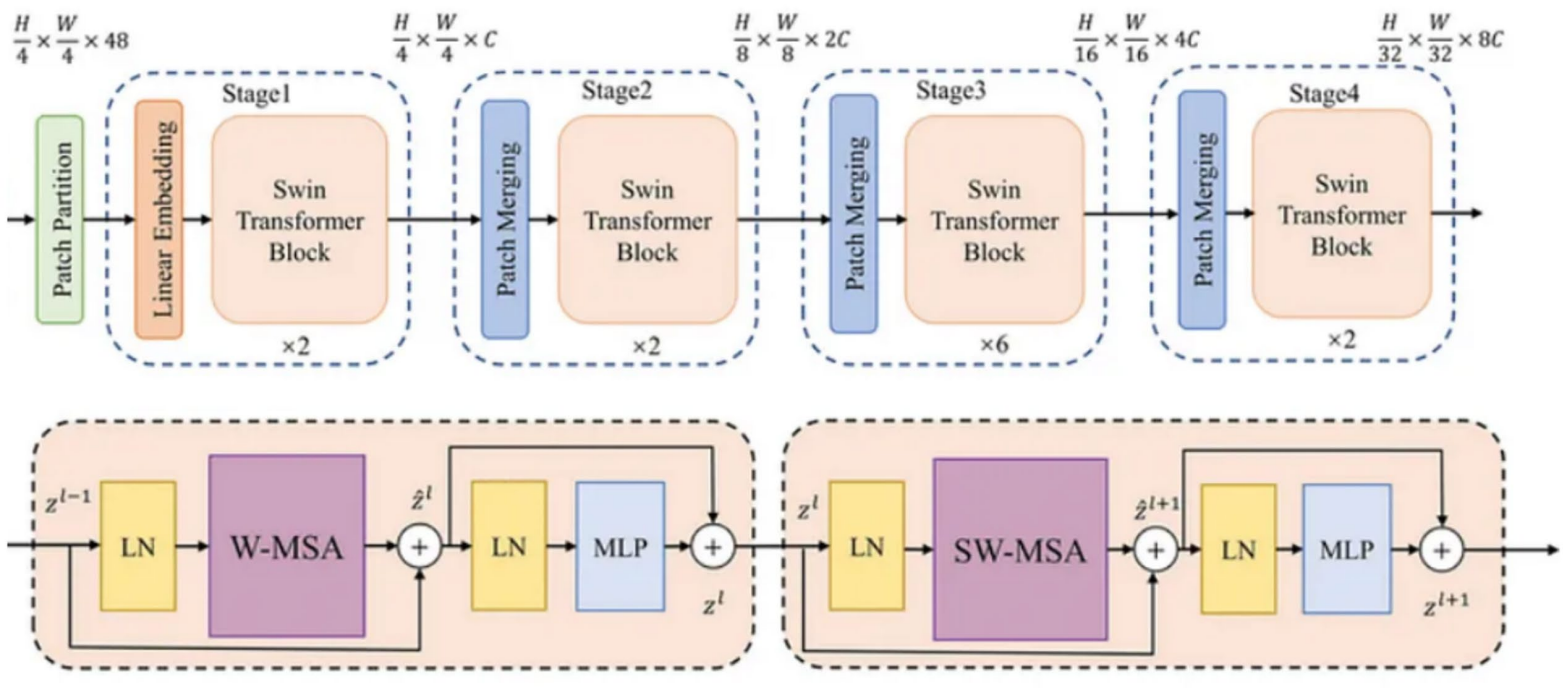
Architecture **(a)** Swin Transformer V2 and **(b)** Swin Transformer Block (Liu et al., 2021).

Swin Transformer V2 is a progressive vision transformer that works on non-overlapping image patches and computes self-attention inside local windows, moving the windows between layers to capture cross-window associations. It addresses the restrictions of worldwide self-attention (e.g., high memory use), while still capturing long-range conditions as shown in Figure 4.

The various architecture components related to this transformer are given below:

- Patch partition

The input image is divided into patches that are non-overlapping. If the input image is of size H × W × 3 and the patch size is 4 × 4, then the output is given in Equation 1.

$$\text{patches}\;\varepsilon \;R^{\frac{H}{4}\times \frac{H}{4}\times (4\times 4\times 3)}$$ (1)

- Linear Embedding

After the patch partition, each patch is projected toward a feature vector with dimension C as shown in Equation 2.

$$x_{o}=\text{patcEmbedding}(x_{\text{patches}})\varepsilon R^{N\times C}$$ (2)

- Window-based multi-head self-attention

Self-attention is calculated within windows as shown in Equation 3. The attention for a patch $x_{i}\in R^{D}$

$$\text{Attention}(Q,K,V)=\text{Soft}\max(\frac{QK^{T}}{\sqrt{d_{k}}}+B)V$$ (3)

Here, Q, K, V are the linear projections of the input, B is the relative position bias that preserves spatial information and d_k_ is the dimensionality of key/query. SwinUNet’s hierarchical attention mechanism and shifted window design give an effective system for capturing both local and global settings, resulting in highly precise and steady segmentation results.

- Shifted Windows
  Every other block shifts the window to enable interaction between windows.
- Hierarchical structure

It merges the patches to downsample as shown in Equation 4.


$$x_{l+1}=\text{PatchMerging}(x_{l})$$ (4)

Owing to its hierarchical design and ability to capture both local and global features efficiently, Swin Transformer V2 emerges as a highly suitable architecture for histopathological image classification in breast cancer diagnosis.

#### 3.2.2 ConvNeXt V2

**Figure 5:**
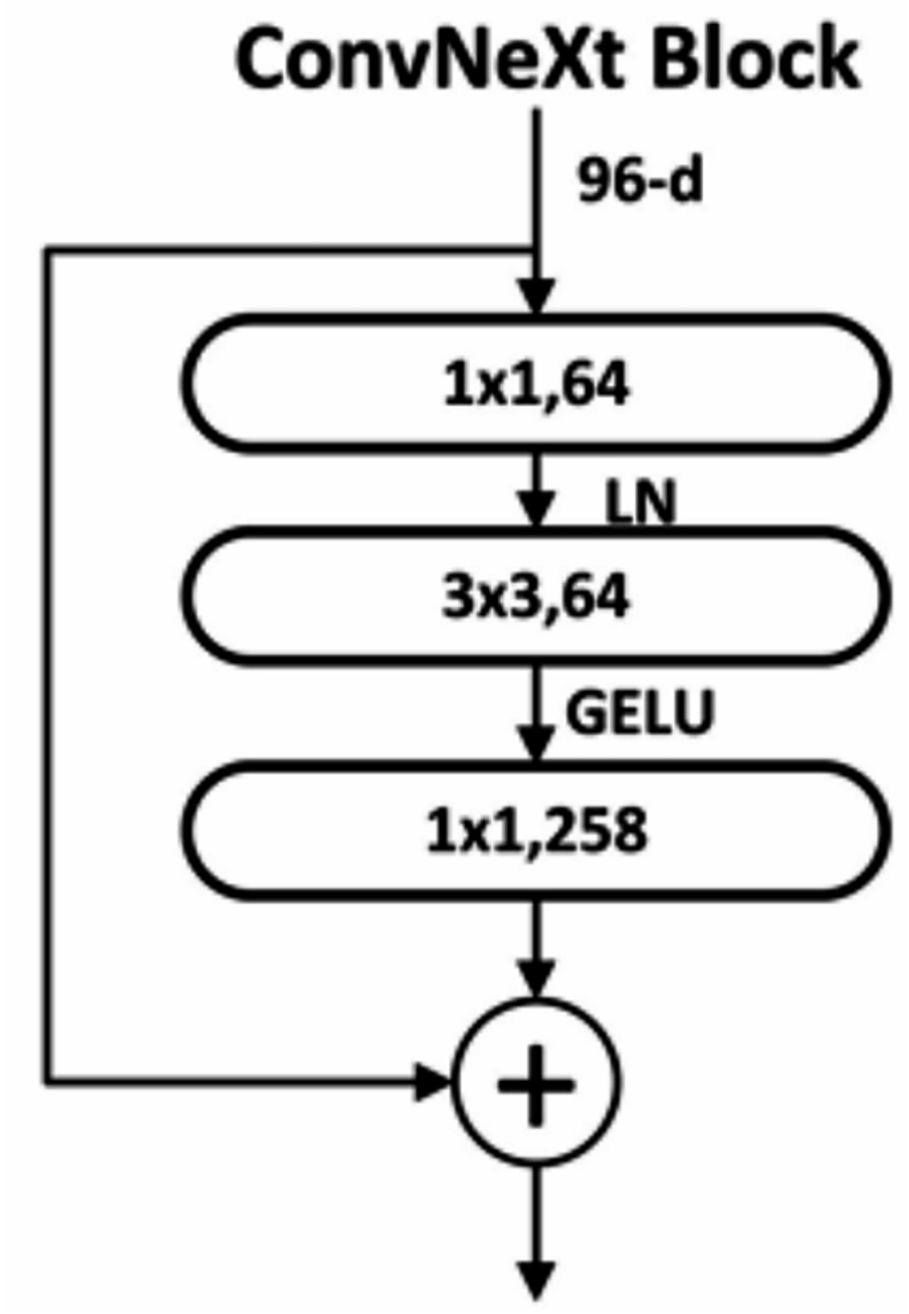
Architecture of ConvNeXt V2 model (Du et al., 2022).

ConvNeXt V2 is a modernized convolutional neural network that consolidates thoughts from transformers (like large kernels and normalization), while retaining the effectiveness and inductive bias of CNNs as shown in Figure 5.

The various architecture components related to this transformer are given below:

- Patchify stem

Convolution acts like a patch embedding as shown in Equation 5 as,

x=Conv2D(x_input,kernel=4,stride=4) (5)

- ConvNeXt block

Each ConvNeXt block consists of Depthwise convolution (DWConv) for spatial mixing, LayerNorm in place of BatchNorm, MLP-like pointwise convolutions, and residual connection shown in Equations 6–8.

x^'=DWConv(x) (6)

y=x+DropPath(x^'') (7)

x_(l+1)=Conv2D(x_l,stride=2) (8)

By integrating modern design principles inspired by vision transformers while retaining the efficiency of convolutional operations, ConvNeXt V2 serves as a competitive baseline for breast cancer classification tasks in histopathological imaging.

## 4 Experiment and results

This section presents the comparative performance analysis of the Swin Transformer V2 and ConvNeXt V2 models on the task of binary breast cancer classification using histopathological images. Various evaluation metrics, including accuracy, were employed to assess the effectiveness of each model.

### 4.1 Epochwise assessment

The displayed Table 3 illustrates the execution measurements of a neural network model over distinctive epochs, giving insight into its learning and generalization capabilities. Here’s a comprehensive summary of the observations in terms of epochs, accuracy (Acc), and loss (Anand et al., 2024; Anand et al., 2025).

**Table 3:** Epochwise results.

Model	Epoch	Loss	Acc	Val_Loss	Val_Acc
Swin Transformer V2	5	0.2448	0.9447	0.2507	0.9390
	10	0.1418	0.9740	0.1576	0.9645
	15	0.0979	0.9828	0.1166	0.9730
	20	0.0732	0.9980	0.0937	0.9850
ConvNeXt V2	5	0.3448	0.7447	0.3207	0.7109
	10	0.3387	0.7805	0.3176	0.7545
	15	0.2793	0.8286	0.2246	0.8009
	20	0.1570	0.8580	0.1937	0.8490

The exploratory observations show that Swin Transformer V2 consistently outperformed ConvNeXt V2 in both training and execution over all epochs. Swin Transformer V2 accomplished higher accuracies with val acc as 93.9% improving to as 98.5% at epoch 20. Swin Transformer V2 illustrated more effective learning with lower training and validation losses throughout. By epoch 20, it achieved a training loss of 0.0732 and a validation loss of 0.0937, whereas ConvNeXt V2 had higher losses of 0.1570 and 0.1937, respectively. Swin Transformer V2’s lower validation loss and higher accuracy show superior generalization on unseen data compared to ConvNeXt V2, which appeared to have slower convergence and a bigger performance gap. The results demonstrate that Swin Transformer V2 gives prevalent performance in terms of both accuracy and loss, making it a more effective model for binary classification of breast cancer in histopathological images.

**Figure 6:**
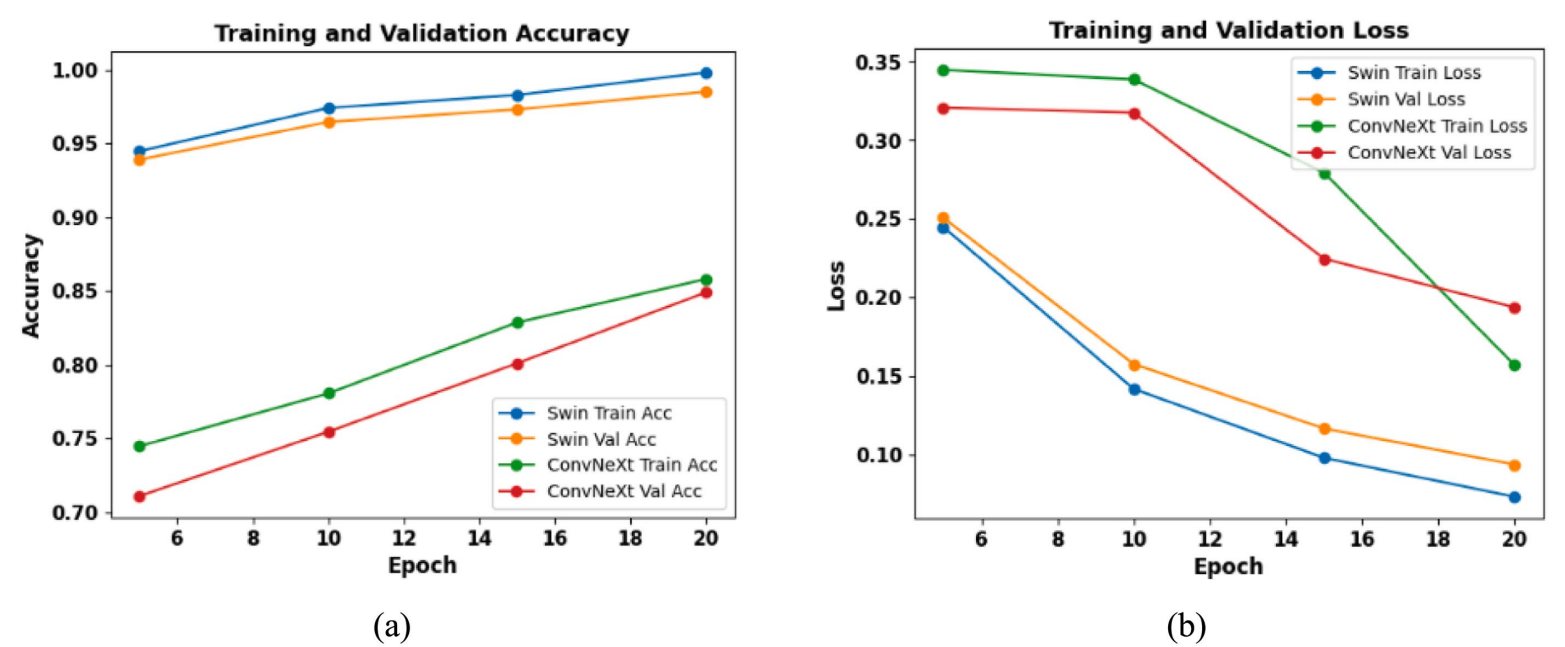
Training and validation for Swin Transformer V2, ConvNeXt V2 **(a)** accuracy, **(b)** loss accuracy and loss are plotted across epochs for both the training and validation sets. Validation performance was used for model selection and early stopping, while final performance metrics are reported on the independent held-out test set.

Figure 6 shows the accuracy and loss curves for Swin Transformer V2 and ConvNeXt V2. Figure 6a shows the training and validation accuracy for Swin Transformer V2 and ConvNext V2. Figure 6b shows the training and validation loss for Swin Transformer V2 and ConvNext V2.

Across 20 training epochs, Swin Transformer V2 demonstrates steady convergence with loss decreasing from 0.2448 to 0.0732 and validation loss from 0.2507 to 0.0937, achieving a final validation accuracy of 98.50%. In contrast, ConvNeXt V2 shows slower improvement, with validation accuracy reaching 84.90% and higher residual loss values. Overall, Swin Transformer V2 exhibits faster convergence, lower loss, and superior generalization performance.

Similarly, Figure 6b illustrates the residual plot for ConvNeXt V2 that shows the red and blue markers to differentiate between the residuals for loss and accuracy. This allows for a visual comparison of the model’s performance on training and validation data across epochs.

When analyzing the effectiveness of a binary classification model, such as one that is intended to differentiate between benign and malignant breast instances, a confusion matrix is an essential instrument to incorporate into the evaluation process. By providing a comprehensive analysis of the model’s predictions, this matrix offers insights into the correctness of the model as well as the error rates it produces.

**Figure 7:**
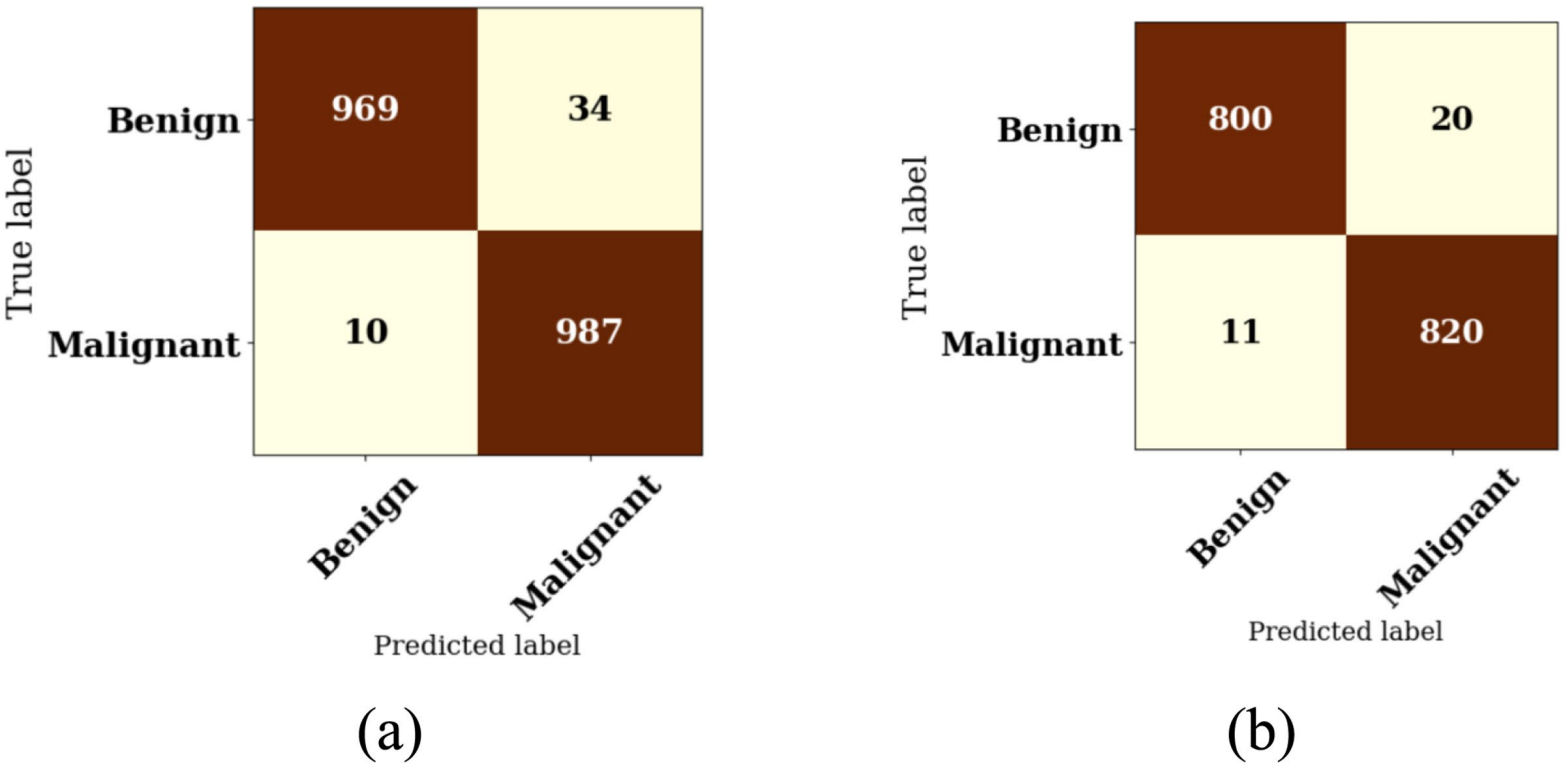
Confusion matrix: **(a)** Swin Transformer V2 and **(b)** ConvNeXt V2. Malignant cases are defined as the positive class. Each matrix reports true positives (TP), true negatives (TN), false positives (FP), and false negatives (FN).

The confusion matrix is a two-by-two table that is two by two and has four key parts as shown in Figure 7. These elements are the true positive (TP), the true negative (TN), the false positive (FP), and the false negative (FN) in the context of breast cancer classification of the disease. Figure 7a depicts the Swin Transformer V2 with Confusion Matrix Values as TP (Malignant correctly predicted) = 987, TN (Benign correctly predicted) = 969, FP (Benign misclassified as Malignant) = 34, FN (Malignant misclassified as Benign) = 10. Figure 7b depicts the ConvNeXt V2 with Confusion Matrix Values as TP = 820, TN = 800, FP = 20, FN = 11.

Those circumstances in which the model successfully identifies malignant instances are referred to as true positives, whereas true negatives indicate that the model accurately recognizes benign occurrences. While false positives occur when benign instances are wrongly categorized as malignant, false negatives occur when malignant cases are incorrectly classified as benign. Both types of errors are considered to be diagnostic errors. Some of the most important performance measures can be generated from these components (Vanacore et al., 2024; Erbani et al., 2024). The model’s execution can be evaluated utilizing key measurements determined from the given confusion matrix values. This shows that when the model predicts a positive class, it is correct 98.9% of the time. In general, these metrics show that the model performs especially well, with high accuracy, precision, recall, and F1 Score.

### 4.2 Analysis with different batch size and optimizer

In this section batch size and optimizers are analyzed on different models.

- Performance on different batch sizes

**Figure 8:**
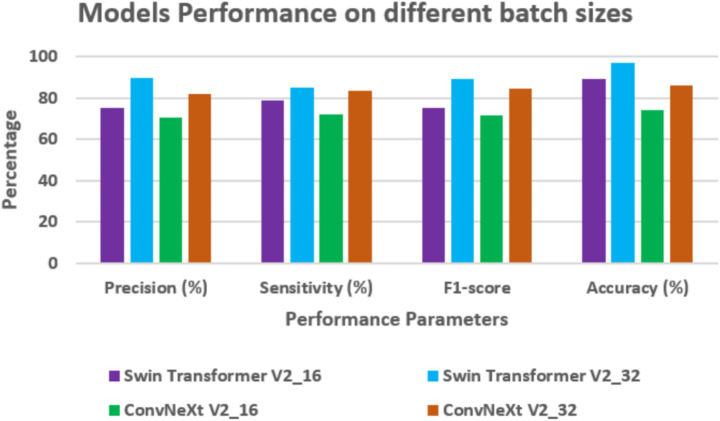
Batchsize assessment on different models.

Table 4 and Figure 8 present the performance of Swin Transformer V2 and ConvNeXt V2 when tested with different batch sizes, 16 and 32, and varying numbers of data samples processed simultaneously. When using a batch size of 16, Swin Transformer V2 did better than ConvNeXt V2 in all four measures. Swin Transformer V2 had a precision of 75.24%, sensitivity of 78.72%, F1-score of 75.20%, and accuracy of 89%. ConvNeXt V2 had lower scores: precision at 70.42%, sensitivity at 72.1%, F1-score at 71.7%, and accuracy at 74%. When the batch size was doubled to 32, both models performed much better. Swin Transformer V2 had even higher results: precision of 89.77%, sensitivity of 85.28%, F1-score of 89.35, and accuracy of 97%. ConvNeXt V2 also improved, but not as much, with precision of 82.14%, sensitivity of 83.4%, F1-score of 84.4%, and an accuracy of 86%.

**Table 4:** Batchsize-wise assessment.

Model	Batch Size	Precision (%)	Sensitivity (%)	F1-score	Accuracy (%)
Swin Transformer V2	16	75.24	78.72	75.20	89
	32	89.77	85.28	89.35	97
ConvNeXt V2	16	70.42	72.1	71.7	74
	32	82.14	83.4	84.4	86

These results exhibit that Swin Transformer V2 is very effective, especially when working with more data per batch. This suggests it’s strong at learning from data and making accurate predictions.

The results also show that the batch size is an important choice when tuning a model, as it can greatly affect how well the model performs.

- Performance on different optimizers

**Figure 9:**
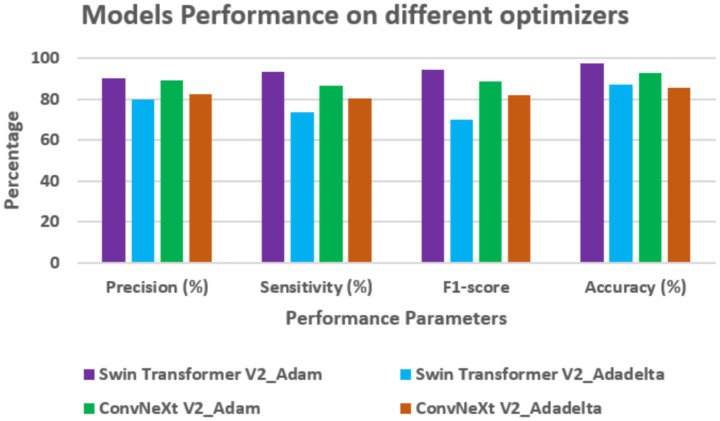
Optimizer-wise assessment on different models.

Table 5 and Figure 9 show how well Swin Transformer V2 and ConvNeXt V2 performed using two different methods called Adam and Adadelta. When using the Adam approach, both models worked better as compared to the case of Adadelta.

**Table 5:** Optimizer-wise assessment.

Model	Optimizer	Precision (%)	Sensitivity (%)	F1-score	Accuracy (%)
Swin Transformer V2	Adam	**90.42**	**93.28**	**94.62**	**97.25**
	Adadelta	79.71	73.57	70.12	87.23
ConvNeXt V2	Adam	89.14	86.42	88.44	93.04
	Adadelta	82.28	80.42	82.16	85.5

Swin Transformer V2 had a precision of 90.42%, sensitivity of 93.28%, F1-score of 94.62%, and an accuracy of 97.25%. ConvNeXt V2 also did well with Adam, with precision at 89.14%, sensitivity at 86.2%, F1-score at 88.44%, and accuracy at 93.04%.

In all these areas, Swin Transformer V2 did better than ConvNeXt V2, showing that it works more effectively with Adam, probably because it learns features better and converges faster. When using Adadelta, both models performed worse. Swin Transformer V2 had a precision of 79.71%, sensitivity of 73.57%, F1-score of 70.12%, and accuracy of 87.23%. ConvNeXt V2 had lower results too with the value of precision as 82.28%, sensitivity as 80.42%, F1-score as 82.16%, and accuracy as 85.5%. All this shows that the choice of method has a big effect on how well the models work. Adam helps both models perform better, especially Swin Transformer V2, which seems to be more affected by the method used. This makes Swin Transformer V2 a good choice for tasks that need high accuracy in computer vision.

### 4.3 Analysis based on splitting ratio

The impact of several dataset splitting ratios—80:20 and 75:25 in particular—on model performance has been examined in this subsection.

- Performance on 80:20 split ratio

**Figure 10:**
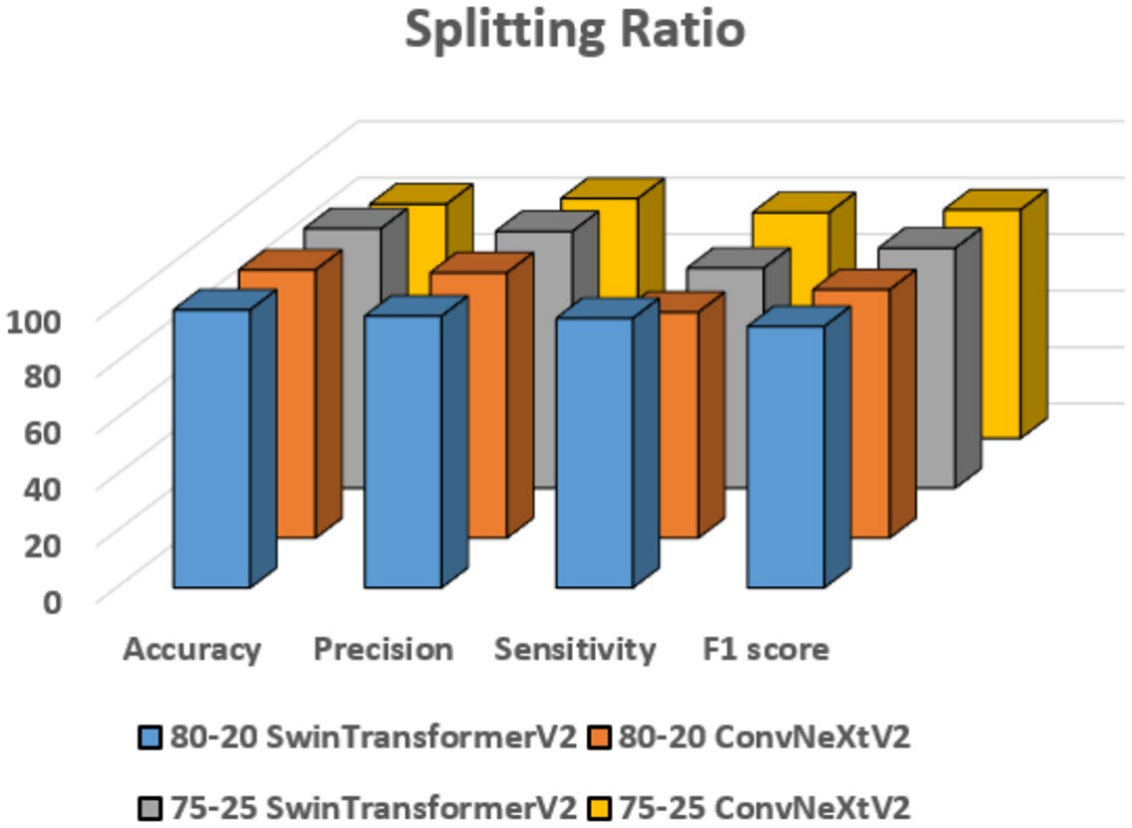
Splitting ratio analysis.

The Swin Transformer V2 model performed better than ConvNeXt V2 in all the tested measures as shown in Table 6 and Figure 10. It had an accuracy of 98.5%, which is much higher than ConvNeXt V2’s 95%. Swin Transformer V2 also had higher precision at 96.3% and sensitivity at 95.5%, showing it is more reliable and better at responding to different cases. Its F1-score of 92.6% shows it has a good balance between precision and recall. On the other hand, ConvNeXt V2 had lower numbers in all areas, with the lowest sensitivity at 80%, which means Swin Transformer V2 gives more consistent and accurate results overall.

- Performance on 75:25 split ratio

**Table 6:** Analysis on 80:20 splitting.

Parameters	SwinTransformerV2	ConvNeXtV2
Accuracy	98.5	95
Precision	96.3	94
Sensitivity	95.5	80
F1 score	92.6	88

The Swin Transformer V2 model exceeded ConvNeXt V2 in most evaluation metrics, show its effectiveness for the given classification task. Swin Transformer V2 attained an accuracy of 92%, notably higher than ConvNeXt V2’s 83%, along with improved precision (91%) and F1-score (85%), indicating better overall reliability and balance between precision and recall as shown in Table 7 and Figure 10. Although ConvNeXt V2 showed slightly higher sensitivity (80%) compared to Swin Transformer V2 (78%), the overall results highlight that Swin Transformer V2 delivers stronger and more stable performance across the majority of metrics.

**Table 7:** Analysis on 75:25 splitting.

Parameters	SwinTransformerV2	ConvNeXtV2
Accuracy	92	83
Precision	91	85
Sensitivity	78	80
F1 score	85	81

### 4.4 Comparison of Swin Transformer V2 and ConvNeXt V2 models

This segment presents a point-by-point comparative examination of the Swin Transformer V2 and ConvNeXt V2 models based on their performance in classifying breast cancer histopathological images as given in Table 8. Both models were assessed over numerous epochs utilizing key execution metrics, including training/validation accuracy and loss, to evaluate their learning effectiveness, generalization capability, and overall classification adequacy.

**Table 8:** Comparison of models.

Aspect	Swin Transformer V2	ConvNeXt V2
Model type	Hierarchical and Window-based Vision Transformer	Convolutional Neural Network
Architecture	Trans former with shifted windows	ResNet-inspired CNN
Feature extraction	Attention-based	Convolution-based
Hierarchical structure	Yes (patch merging across layers)	Yes (stage-wise downsampling)
Best use case	Large datasets, complex spatial dependencies	Small-to-medium datasets, faster prototyping
Attention mechanism	Window Multi-head Self-Attention (W-MSA)	No attention; uses depthwise separable convolutions
Normalization	LayerNorm (in transformer blocks)	LayerNorm (channels-last)
Residual connections	Yes (inside transformer blocks)	Yes (inside ConvNeXt blocks)
MLP/FFN structure	Two-layer MLP in attention blocks	MLP-style pointwise convolutions
Pretrained weights	ImageNet-1K	ImageNet-1K
Optimizer	Adam	Adam
LR scheduler	Cosine/Step	Cosine/Step
Batch size	16, 32	16, 32
Epochs	20	20

To ensure a fair architectural comparison, both Swin Transformer V2 and ConvNeXt V2 were trained under identical experimental conditions. All hyperparameters, optimization strategies, and data augmentation techniques were matched across models to eliminate tuning bias.

Performance across five independent random seeds demonstrates strong stability and reproducibility for Swin Transformer V2, with a mean accuracy of 98.5 ± 0.15%, precision of 96.3 ± 0.15%, sensitivity of 95.5 ± 0.23%, and F1-score of 92.7 ± 0.19% is shown in Table 9. The low standard deviation indicates minimal variance across runs. In comparison, ConvNeXt V2 shows consistently lower performance, particularly in sensitivity (~80%), highlighting weaker malignant detection capability. Overall, Swin Transformer V2 exhibits superior robustness, convergence stability, and generalization across multiple initialization seeds.

**Table 9:** Performance across five random seeds.

Seed	Model	Accuracy (%)	Precision (%)	Sensitivity (%)	F1-score (%)
42	Swin Transformer v2	98.4	96.2	95.3	92.4
	Convnext v2	94.8	93.8	79.5	87.6
123	Swin Transformer v2	98.6	96.5	95.7	92.8
	Convnext v2	95.2	94.1	80.2	88.3
2024	Swin Transformer v2	98.5	96.3	95.6	92.7
	Convnext v2	95.0	94.0	80.0	88.1
7	Swin Transformer v2	98.3	96.1	95.2	92.5
	Convnext v2	94.9	93.9	79.8	87.9
99	Swin Transformer v2	98.7	96.4	95.8	92.9
	Convnext v2	95.1	94.2	80.2	88.4
Mean ± STD		98.5 ± 0.15	96.3 ± 0.15	95.5 ± 0.23	92.7 ± 0.19

### 4.5 Comparison of best performing Swin Transformer V2 model with other models

In this section, a comparison of three models, namely ViT (Vision Transformer), DeiT (Data-efficient Image Transformer), and BEiT (Bidirectional Encoder representation from Image Transformers), is performed as given in Table 10.

**Table 10:** Comparison of best model with other models.

Model	Architecture	Pre-training strategy	Strengths	Weaknesses
ViT (Aburass et al., 2025)	Transformer	Supervised on large data	Global feature modelling	Requires large data
DeiT (Tang et al., 2025)	Transformer	Distillation and supervised	Better on small data	Less capacity than ViT
BEiT (Baboshina et al., 2025)	Transformer	Masked image modeling + supervised fine tuning	Strong on subtle textures	More complex pre-training
Swin	Hierarchical Transformer	Supervised or self-supervised	Local and Global feature modelling	High computational complexity

### 4.6 Visualization of results

**Figure 11:**
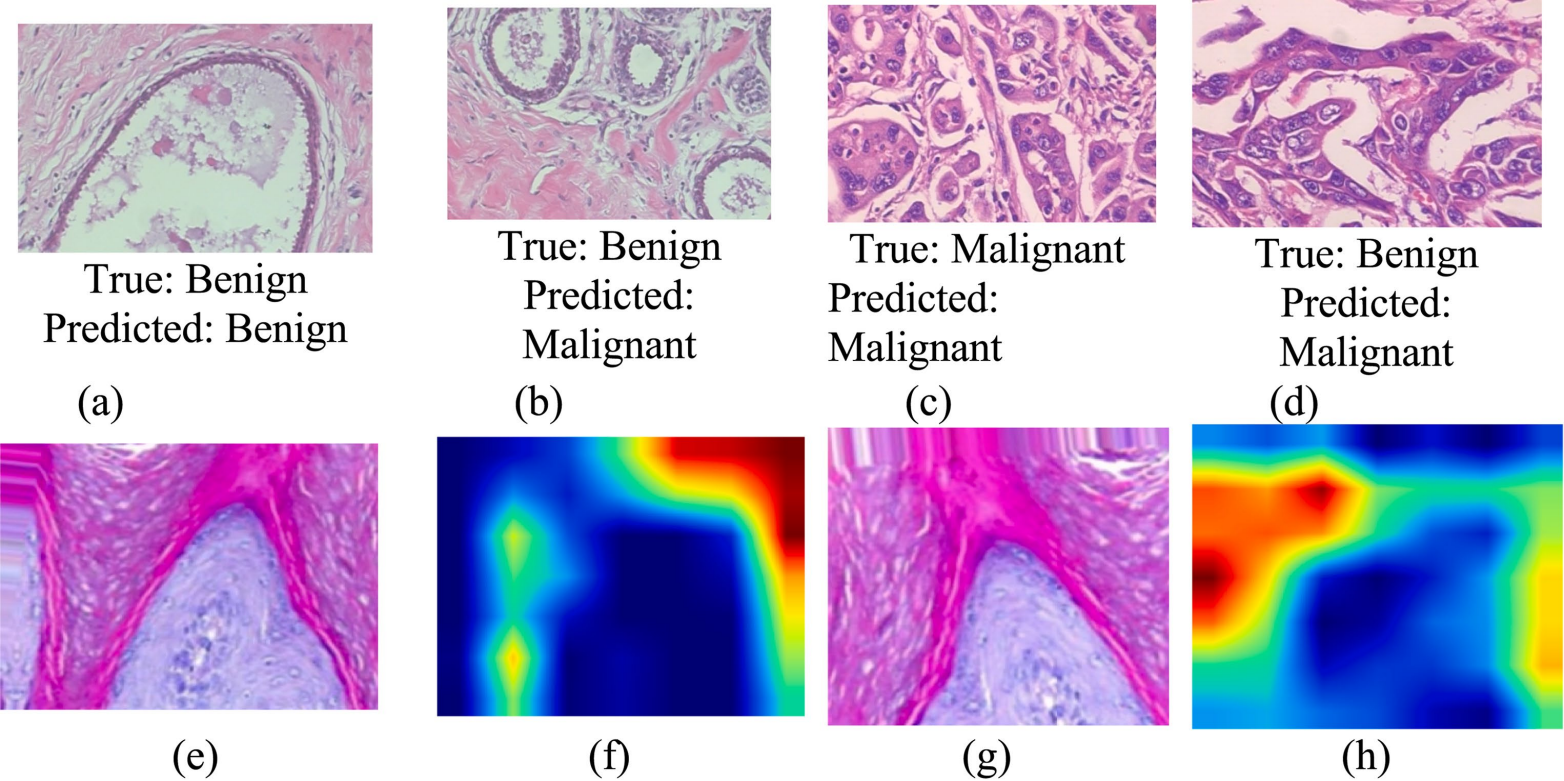
Visualization **(a,c)**, classification **(b,d)**, misclassification **(e,g)**, original image **(f,h)**, GradCAM image.

Figure 11 shows the visualization results, such as true and predicted images for breast cancer.

Classification and misclassification in benign and malignant breast cancer occur due to several factors related to data quality, model limitations, and inherent challenges in distinguishing between the two categories. A model can accurately classify benign and malignant breast cancer when benign and malignant tumors have well-defined characteristics in imaging (e.g., size, shape, texture) and pathology (e.g., cellular structure, gene expression). Important biomarkers and radiological features (such as margins, density, or microcalcifications) are considered, leading to better predictions. Misclassification can happen due to several reasons. Some benign tumors may appear similar to malignant ones (e.g., atypical hyperplasia) and vice versa, leading to confusion. Low-resolution images, poor staining in pathology slides, or mislabeled samples can introduce errors. If one class (e.g., benign cases) is more frequent in the dataset, the model might be biased toward predicting that class more often. If the model is too complex, it may memorize noise instead of generalizing patterns (overfitting). If it’s too simple, it may fail to capture critical differences (underfitting). Different imaging techniques, patient demographics, or slight variations in tumor structure may confuse. To improve classification accuracy, techniques such as data augmentation, feature engineering, better model selection, and the inclusion of expert medical knowledge are often used.

The Grad-CAM (Gradient-weighted Class Activation Mapping) technique was applied to visualize the regions of breast histopathological images that most influenced the CNN’s classification decisions. Using the generate_gradcam_heatmap function, heatmaps were generated by computing the gradient of the predicted class score with respect to the last convolutional layer. These heatmaps were superimposed on the original images, highlighting discriminative areas that guided the model’s decision-making, thus enhancing interpretability.

### 4.7 State-of-the art comparison

In recent studies, different deep learning and machine learning methods are explored for classifying breast cancer using images of histopathology as given in Table 11.

**Table 11:** State-of-the-art.

Ref/Year	Technique	Dataset/No. of Images	Accuracy
Yan et al. (2025)	Neighborhood attention transformer	BACH/605	91.25%
Karthiga et al. (2025)	Deep belief network	BreakHis/9,109	96%
Gupta et al. (2025)	Support vector classifier	BreakHis/9,109	90.17%
Yusuf et al. (2025)	Deep CNN	BreakHis/7,909	96%
Abimouloud et al. (2025)	Combination of CNN and ViT	BreakHis/7,925	97.02%
Korkmaz and Kaplan (2025)	CNN	Kaggle/1,116	93.80%
Ain et al. (2025)	Genetic programming feature learning	BreakHis/7,909	76.98%
Our	Swin Transformer V2	Breast cancer/10,000	98.5%

For example, the Neighborhood Attention Transformer (Yan et al., 2025) got a 91.25% accuracy on the BACH dataset, which has 605 images. This shows that attention mechanisms can be helpful in this area. Deep belief networks (Karthiga et al., 2025) and deep CNNs (Yusuf et al., 2025), trained on the BreakHis dataset with over 9,000 images, achieved accuracies of 96%, showing that deep hierarchical feature extraction works well. Traditional methods like support vector classifiers (Gupta et al., 2025) scored lower, at 90.17% on BreakHis, suggesting they aren’t as good at catching complex patterns in histology. Other techniques, such as a CNN trained on a smaller Kaggle dataset (Korkmaz and Kaplan, 2025) achieved 93.80%, and genetic programming-based feature learning (Ain et al., 2025) performed worse with 76.98%.

**Figure 12:**
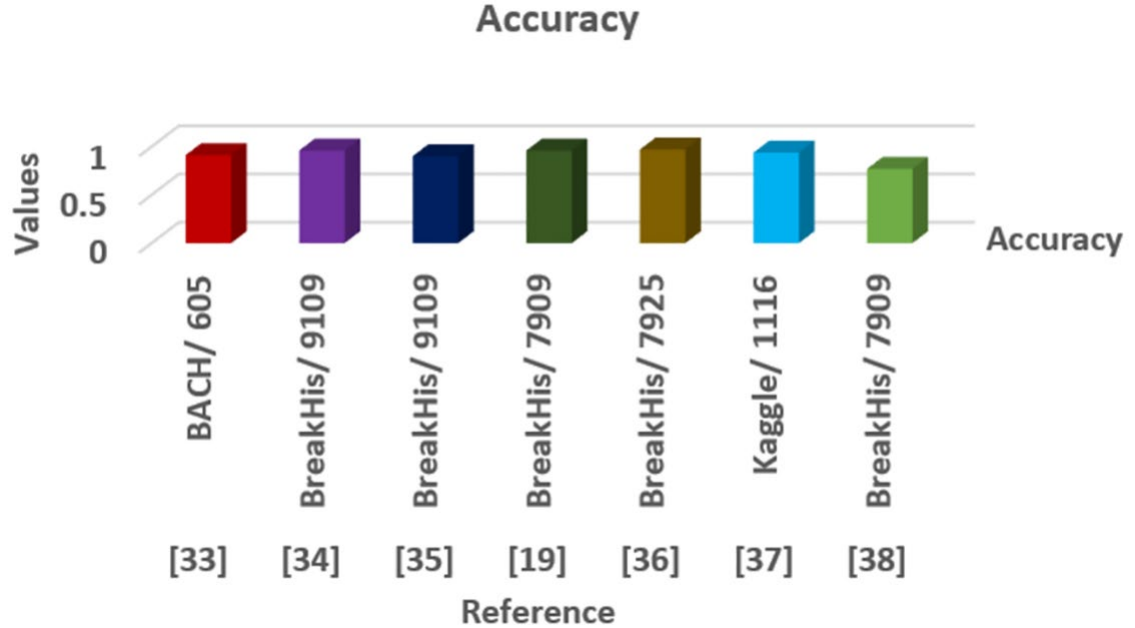
State-of-the-art comparison.

The proposed approach, which uses the Swin Transformer V2, performs much better than these methods. It achieved 98.5% accuracy on a bigger and more varied dataset of 10,000 high-resolution histopathological images as shown in Figure 12.

This shows that hierarchical transformer architectures, especially with shifted window attention, are strong at capturing both fine details and overall context. This makes them better at accurately and reliably classifying breast cancer.

## 5 Discussion

The comparative analysis between Swin Transformer V2 and ConvNeXt V2 demonstrates that transformer-based architectures hold substantial potential for digital pathology applications. The superior performance of Swin Transformer V2, achieving an accuracy of 0.985, can be attributed to its shifted window self-attention mechanism, which effectively captures both local texture patterns and long-range contextual dependencies in histopathological images. In contrast, ConvNeXt V2, while efficient in modeling hierarchical spatial features through convolutional operations, is relatively constrained in representing non-local relationships that are often crucial for distinguishing subtle morphological differences between benign and malignant tissues. This distinction underlines the capability of attention-driven models to learn complex spatial correlations that traditional convolutional structures may overlook.

Despite the encouraging results, this study has certain limitations. The dataset, although containing 10,000 high-resolution images, may not comprehensively represent the full variability encountered in clinical settings, such as differences in staining quality, tissue preparation, and scanner characteristics. Moreover, transformer-based architectures like Swin Transformer V2 are computationally intensive, requiring high memory and processing resources for fine-tuning and inference, which could limit their deployment in resource-constrained clinical environments.

In recent years, several advanced deep learning frameworks have been proposed for breast cancer histopathological image classification. Abdulwahhab et al. (2025) introduced HAFMAB-Net, a hierarchical dual-path architecture that integrates multilevel attention-enhanced bottleneck blocks with adaptive feature fusion. By effectively capturing both global contextual information and fine-grained spatial features, the model achieved high classification accuracy on the BACH, BreaKHis, and LC25000 datasets, demonstrating strong robustness in both binary and multi-class settings.

Similarly, Abdulaal et al. (2025) proposed Mega Ensemble Net (MEMF-Net), an ensemble-based framework that combines multi-scale patch-level and full-image features using fine-tuned ResNet-18, ResNet-34, and ResNet-50 models. Through transfer learning, patch-based augmentation, and Random Forest-based feature fusion, the method enhances feature diversity and generalization capability. Evaluated on the BACH and BreaKHis datasets with four-fold cross-validation, MEMF-Net achieved up to 99% test accuracy, while effectively mitigating overfitting.

In contrast, Abdulaal et al. (2024) presented a hybrid diagnostic framework that integrates unsupervised and supervised learning strategies on the BreaKHis dataset. A pre-trained VGG19 model was employed for feature extraction from sub-images, while a Variational Autoencoder (VAE) and k-means clustering were used to learn latent representations and identify intrinsic data groupings. The clustered features were subsequently classified using a VGG19-SVM model. This combined representation learning and supervised classification approach achieved 98.56% accuracy at 100 × magnification, highlighting the effectiveness of integrating unsupervised learning with deep feature extraction.

Overall, these studies collectively demonstrate that attention mechanisms, ensemble strategies, and hybrid unsupervised–supervised frameworks significantly enhance classification performance in breast cancer histopathological image analysis.

Although the proposed framework demonstrates strong performance on the evaluated dataset, external validation across multiple institutions and diverse acquisition settings is necessary to confirm its generalizability. Future studies will focus on multi-center evaluation and prospective clinical testing to assess real-world applicability. Future work will focus on addressing these limitations by extending the study to multi-class classification of breast cancer subtypes and incorporating larger and more diverse datasets to enhance generalizability. Additionally, integrating interpretability techniques such as Gradient-weighted Class Activation Mapping (Grad-CAM) or attention heatmaps will provide visual insights into the regions that most influence model predictions, thereby improving transparency and trust in AI-assisted diagnosis. Further exploration into hybrid transformer-convolutional frameworks may also yield efficient yet powerful solutions for digital pathology.

## 6 Conclusion

In this research, a deep learning-based approach was executed for the binary classification of breast cancer utilizing high-resolution histopathological images. Two advanced vision architectures—Swin Transformer V2 and ConvNeXt V2—were fine-tuned and assessed for their viability in recognizing between benign and malignant cases. The experimental results indicate that Swin Transformer V2 beats ConvNeXt V2 over all key metrics.

- Swin Transformer V2 consistently outperformed ConvNeXt V2, achieving higher validation accuracy (98.5% vs. 84.9%) and lower validation loss (0.0937 vs. 0.1937) by epoch 20.
- While Swin Transformer V2 demonstrated rapid convergence, ConvNeXt V2 showed slower improvement in both accuracy and loss across all epochs.

By decreasing the risk of human mistakes and assisting in decision-making, this approach has the potential to improve patient results altogether. However, the model illustrates solid execution; further approval in assorted clinical situations is required to evaluate its strength over diverse demographics and imaging conditions. Integration with radiological and genomic information could improve diagnostic accuracy. Generally, this research marks a noteworthy step toward breast cancer determination, pointing to improved accuracy and proficiency in medical decision-making. The future work is as follows:

- Integrate larger and more diverse datasets to progress the model’s adaptability across different imaging conditions.
- Integrate additional imaging techniques such as mammograms and MRIs to provide a more comprehensive diagnostic approach.
- Develop interpretability methods to help clinicians understand and trust AI-driven decisions.
- Optimize the model for real-world applications by integrating it into hospital workflows.

Connect the AI model with patient records for seamless diagnostic support and better decision-making.

## Data Availability

The original contributions presented in the study are included in the article/supplementary material, further inquiries can be directed to the corresponding author.

## References

[ref1] Abdulaal A. H. Abdulwahhab A. H. Breesam A. M. Oleiwi Z. H. Yassin R. A. Valizadeh M. . (2025). MEMF-net: a mega-ensemble of multi-feature CNNs for classification of breast histopathological images. Iraqi J. Comput. Sci. Math. 6:36. doi: 10.52866/2788-7421.1309

[ref2] Abdulaal A. H. Valizadeh M. Yassin R. A. Albaker B. M. Abdulwahhab A. H. Amirani M. C. . (2024). Unsupervised histopathological sub-image analysis for breast cancer diagnosis using variational autoencoders, clustering, and supervised learning. J. Eng. Sustain. Dev. 28, 729–744. doi: 10.31272/jeasd.28.6.6

[ref3] Abdulwahhab A. H. Bayat O. Ibrahim A. A. (2025). HAFMAB-net: hierarchical adaptive fusion based on multilevel attention-enhanced bottleneck neural network for breast histopathological cancer classification. Signal Image Video Process 19:410. doi: 10.1007/s11760-025-04001-1

[ref4] Abimouloud M. L. Bensid K. Elleuch M. Ammar M. B. Kherallah M. (2025). Advancing breast cancer diagnosis: token vision transformers for faster and accurate classification of histopathology images. Vis. Comput. Ind. Biomed. Art 8:1. doi: 10.1186/s42492-024-00181-8, 39775534 PMC11711433

[ref5] Aburass S. Dorgham O. Al Shaqsi J. Abu Rumman M. Al-Kadi O. (2025). Vision transformers in medical imaging: a comprehensive review of advancements and applications across multiple diseases. J. Imaging Inform. Med. 38, 3928–3971. doi: 10.1007/s10278-025-01481-y, 40164818 PMC12701147

[ref6] Ain Q. U. Al-Sahaf H. Xue B. Zhang M. (2025). Genetic programming for malignancy diagnosis from breast cancer histopathological images: a feature learning approach. IEEE Trans. Emerg. Top. Comput. Intell., 1–14. doi: 10.1109/tetci.2024.3523769

[ref7] Anand V. Khajuria A. Pachauri R. K. Gupta V. (2025). Optimized machine learning based comparative analysis of predictive models for classification of kidney tumors. Sci. Rep. 15:30358. doi: 10.1038/s41598-025-15414-w, 40830637 PMC12365197

[ref8] Anand V. Koundal D. Alghamdi W. Y. Alsharbi B. M. (2024). Smart grading of diabetic retinopathy: an intelligent recommendation-based fine-tuned EfficientNetB0 framework. Front. Artif. Intell. 7:1396160. doi: 10.3389/frai.2024.1396160, 38694880 PMC11062181

[ref11] Baboshina V. A. Lyakhov P. A. Lyakhova U. A. Pismennyy V. A. (2025). Bidirectional encoder representation from image transformers for recognizing sunflower diseases from photographs. Computer 49:3. doi: 10.18287/2412-6179-CO-1514

[ref12] CBIS-DDSM. (2017) Curated Breast Imaging Subset of Digital Database for Screening Mammography. Available online at: https://www.cancerimagingarchive.net/collection/cbis-ddsm/

[ref9] Ceylan E. (2025). Available online at: https://www.kaggle.com/datasets/emirhanceylan/mammography-breast-cancer-detection (Accessed October 12, 2025).

[ref13] Cheng J.-Z. Ni D. Chou Y.-H. Qin J. Tiu C.-M. Chang Y.-C. . (2016). Computer aided diagnosis with deep learning architecture: applications to breast lesions in us images and pulmonary nodules in CT scans. Sci. Rep. 6:24454. 27079888 10.1038/srep24454 PMC4832199

[ref14] Du H. Zhu W. Peng K. Li W. (2022). Improved high speed flame detection method based on YOLOv7. Open J. Appl. Sci. 12, 2004–2018. doi: 10.4236/ojapps.2022.1212140

[ref15] Erbani J. Portier P. É. Egyed-Zsigmond E. Nurbakova D. (2024). Confusion matrices: a unified theory. IEEE Access 12, 181372–181419. doi: 10.1109/ACCESS.2024.3507199

[ref16] Fiaz A. Raza B. Faheem M. Raza A. (2024). A deep fusion-based vision transformer for breast cancer classification. Healthc. Technol. Lett. 11, 471–484. doi: 10.1049/htl2.12093, 39720758 PMC11665795

[ref17] Fujioka T. Kubota K. Mori M. Kikuchi Y. Katsuta L. Kasahara M. . (2019). Distinction between benign and malignant breast masses at breast ultrasound using deep learning method with convolutional neural network. Jpn. J. Radiol. 37, 466–472. doi: 10.1007/s11604-019-00831-5, 30888570

[ref18] Gupta M. Verma N. Sharma N. Singh S. N. Brojen Singh R. K. Sharma S. K. (2025). Deep transfer learning hybrid techniques for precision in breast cancer tumor histopathology classification. Health Informat. Sci. Syst. 13:20. doi: 10.1007/s13755-025-00337-7, 39949707 PMC11813847

[ref19] Hossain M. S. Rahman A. Ahmed M. Fatema K. Mahbubul M. M. (2025). Vision transformer for the categorization of breast Cancer from H&E Histopathology Images. J. Image Graph. 13. doi: 10.18178/joig.13.4.380-393

[ref20] Huang Q. Huang Y. Luo Y. Yuan F. Li X. (2020). Segmentation of breast ultrasound image with semantic classi_-cation of superpixels. Med. Image Anal. 61:101657. doi: 10.1016/j.media.2020.101657 32032899

[ref21] Huynh B. Drukker K. Giger M. (2016). Mo-de-207b-06: computer-aided diagnosis of breast ultrasound images using transfer learning from deep convolutional neural networks. Med. Phys. 43:3705. doi: 10.1118/1.4957255

[ref22] Javed H. El-Sappagh S. Abuhmed T. (2025). Robustness in deep learning models for medical diagnostics: security and adversarial challenges towards robust AI applications. Artif. Intell. Rev. 58, 1–107.

[ref23] Jiang B. Bao L. He S. Chen X. Jin Z. Ye Y. (2024). Deep learning applications in breast cancer histopathological imaging: diagnosis, treatment, and prognosis. Breast Cancer Res. 26:137. doi: 10.1186/s13058-024-01895-6, 39304962 PMC11416021

[ref24] Kaczmarek M. Kowal M. Korbicz J. (2025). Exploring data preparation strategies: a comparative analysis of vision transformer and ConvNext architectures in breast Cancer histopathology classification. Int. J. Appl. Math. Comput. Sci. 35, 329–339. doi: 10.61822/amcs-2025-0023

[ref25] Karthiga R. Narasimhan K. Raju N. Amirtharajan R. (2025). Automatic approach for breast cancer detection based on deep belief network using histopathology images. Multimed. Tools Appl. 84, 4733–4750.

[ref26] Korkmaz M. Kaplan K. (2025). Effectiveness analysis of deep learning methods for breast cancer diagnosis based on histopathology images. Appl. Sci. 15:1005. doi: 10.3390/app15031005

[ref27] Lee H. Park J. Hwang J. Y. (2020). Channel attention module with multiscale grid average pooling for breast cancer segmentation in an ultrasound image. IEEE Trans. Ultrason. Ferroelectr. Freq. Control 67, 1344–1353. 32054578 10.1109/TUFFC.2020.2972573

[ref28] Liu Z. Lin Y. Cao Y. Hu H. Wei Y. Zhang Z. . (2021). Swin transformer: hierarchical vision transformer using shifted windows. In Proceedings of the IEEE/CVF International Conference on Computer vision (pp. 10012–10022).

[ref10] Naren OS. (2024). Available online at: https://www.kaggle.com/datasets/obulisainaren/multi-cancer/data

[ref29] Pandey S. K. Rathore Y. K. Ojha M. K. Janghel R. R. Sinha A. Kumar A. (2025). BCCHI-HCNN: breast cancer classification from histopathological images using hybrid deep CNN models. J. Imaging Informatics Med. 38, 1690–1703. doi: 10.1007/s10278-024-01297-2, 39402357 PMC12092882

[ref30] Pradeepa M. Sharmila B. Nirmala M. (2025). A hybrid deep learning model EfficientNet with GRU for breast cancer detection from histopathology images. Sci. Rep. 15:24633. doi: 10.1038/s41598-025-00930-6, 40634313 PMC12241594

[ref31] Shan J. Cheng H. Wang Y. (2012). Completely automated segmentation approach for breast ultrasound images using multiple-domain features. Ultrasound Med. Biol. 38, 262–275. doi: 10.1016/j.ultrasmedbio.2011.10.022, 22230134

[ref32] Shiri M. Reddy M. P. Sun J. (2024). Supervised Contrastive vision Transformer for Breast Histopathological Image Classification. In 2024 IEEE International Conference on Information Reuse and Integration for Data Science (IRI) (pp. 296–301). IEEE.

[ref33] Springenberg M. Frommholz A. Wenzel M. Weicken E. Ma J. Strodthoff N. (2023). From modern CNNs to vision transformers: assessing the performance, robustness, and classification strategies of deep learning models in histopathology. Med. Image Anal. 87:102809. doi: 10.1016/j.media.2023.102809, 37201221

[ref34] Tang H. Liu D. Shen C. (2025). Data-efficient multi-scale fusion vision transformer. Pattern Recogn. 161:111305. doi: 10.1016/j.patcog.2024.111305

[ref35] Torbati N. Ayatollahi A. Kermani A. (2014). An efficient neural network based method for medical image segmentation. Comput. Biol. Med. 44, 76–87. doi: 10.1016/j.compbiomed.2013.10.029 24377691

[ref36] Vanacore A. Pellegrino M. S. Ciardiello A. (2024). Fair evaluation of classifier predictive performance based on binary confusion matrix. Comput. Stat. 39, 363–383. doi: 10.1007/s00180-022-01301-9

[ref37] Wakili M. A. Shehu H. A. Abdollahi M. Imam Y. U. B. Sharif M. H. Kusetogullari H. (2025). DenseNet-FPA: integrating DenseNet and flower pollination algorithm for breast Cancer histopathology image classification. IEEE Access 13, 145828–145848. doi: 10.1109/access.2025.3599319

[ref38] Yan Y. Lu R. Sun J. Zhang J. Zhang Q. (2025). Breast cancer histopathology image classification using transformer with discrete wavelet transform. Med. Eng. Phys. 138:104317. doi: 10.1016/j.medengphy.2025.104317 40180530

[ref39] Yap M. H. Pons G. Marti J. Ganau S. Sentis M. Zwiggelaar R. . (2017). Automatedbreast ultrasound lesions detection using convolutional neural networks. IEEE J. Biomed. Health Inform. 22, 1218–1226. 28796627 10.1109/JBHI.2017.2731873

[ref40] Yusuf M. Kana A. F. D. Bagiwa M. A. Abdullahi M. (2025). Multi-classification of breast cancer histopathological image using enhanced shallow convolutional neural network. J. Eng. Appl. Sci. 72:24. doi: 10.1186/s44147-025-00589-w

